# African jewel fish (*Hemichromis bimaculatus*) distinguish individual faces based on their unique iridophore patterns

**DOI:** 10.1007/s10071-023-01790-1

**Published:** 2023-06-03

**Authors:** Richard G. Coss, Carol Lee Tyler

**Affiliations:** 1 Department of Psychology, University of California, Davis, CA 95616 USA; 2 Department of Psychology, University of California, Davis, CA USA; 3 807 Falcon Avenue, Davis, CA 95616 USA; 4 1313 West Hellman Ave, Alhambra, CA 91803 USA

**Keywords:** Individual recognition, Face models, Habituation, *Hemichromis bimaculatus*, Iridophore patterns, Jewel fish, Respiration rate

## Abstract

Previous research has shown that African jewel fish (*Hemichromis bimaculatus*) recognize pair-bonded mates during their exchanges of egg-guarding duties. The current research examined the perceptual cues for face recognition by comparing two face models displaying anatomically realistic arrangements of blue iridophores derived from discriminant function analysis of distinct sibling groups. Four groups each consisting of 9 subadults were examined using a narrow compartment restraining lateral movement where face models were presented at eye level for eight trials. Because respiratory movement of the operculum can mechanically displace the eye thereby shifting the retinal image, jewel fish reduce their respiration rate during increased attention. When two experimental groups were presented with the same face models on four trials following initial model presentations, both groups exhibited stable respiration rates indicative of model habituation. When the habituated face models were switched to novel face models on the fifth trial, the rates of respiration decreased as measured by reliable increases in the elapsed times of opercular beats. Switching the models back to the habituated models on the sixth trial caused reliable decreases in the elapsed times of opercular beats, resembling the earlier trials for the habituated models. Switching the face models again to the formerly novel models on the seventh trial produced respiration rates that resembled those of the habituated models. The two control groups viewing the same models for all eight trials exhibited no substantial change in respiration rates. Together, these findings indicate that jewel fish can learn to recognize novel faces displaying unique arrangements of iridorphores after one trial of exposure.

**Supplementary Information:**

The online version contains supplementary material available at 10.1007/s10071-023-01790-1.

## Introduction

Individual face recognition is an essential component of face-to-face social interactions that has received renewed attention in the literature (e.g., Tibbetts and Dale 2007; Wiley 2013; Kohda et al. 2023). The current experimental research examines the individual face-recognition ability of a highly territorial cichlid, the African jewel fish (*Hemichromis bimaculatus* (Gill 1862) that establishes stable pair bonds ensuring cooperative nest defense (see Noble and Curtis 1939). As a more refined visual component of individual recognition, individual face recognition requires sufficient visual acuity to distinguish the facial features of individuals at close proximity. Whereas some teleost fish have evolved the specialized visual ability to seemingly distinguish conspecifics based on general body features (e.g., Balshine-Earn and Lotem 1998), only a few studies have identified the specific facial features evaluated during the process of individual recognition, relying mostly on behavioral evidence suggestive of individual recognition. In this context, for example, individual recognition by the trout (*Oncorhynchus mykiss*) is inferred from staged dominance contests in which the recognition memory of previous encounters reduces subsequent aggressive behavior (Johnsson 1997).

Individual recognition has been documented based on schooling preferences in wild guppies (*Poecilia reticulata*), a perceptual ability constrained by schooling group sizes (Griffiths and Magurran 1997). In another experimental study, bluegill sunfish (*Lepomis macrochirus*) showed evidence of individual recognition inferred from their time spent with familiar vs. unfamiliar individuals (Brown and Colgan 1986). Olfactory cues, however, might have facilitated individual recognition as has been found for zebrafish (*Danio rerio*) using a similar familiar vs. unfamiliar fish exposure protocol (Madeira and Oliveira 2017).

More direct evidence of individual recognition based on the head region, but not frontal views, has emerged with the study of the lyretail cichlid (*Neolamprologus brichardi*), a species that engages in brood defense using individually recognized helpers. In the context of brood defense against egg predators, *N. brichardi* needs to identify quickly the visual cues of familiar helpers, possibly using variation in the intensity of the horizontal bar pattern spanning from the eye to the elongated spot at the opercular (gill cover) rim (Hert 1985). This supposition that opercular bar or stripe patterns might act as a kin-recognition cue was further explored by Le Vin (2011), who also considered that these facial stripes and the intensity of color surrounding these stripes might foster conspecific recognition and influence mate choice for *N. brichardi*. Hert’s (1985) supposition about the importance of the facial patterns of *N. brichardi* profiles acting as individual-recognition cues received strong support from Kohda and colleagues (2015) who used videos of fish with enhanced facial patterns to study individual recognition in a related species, *N. pulcher*. In their research, videos of the side views of familiar neighbors and unfamiliar fish were presented to percipients and the duration of watching was measured. Familiar fish engendered a reliably shorter duration of watching than did the unfamiliar fish, supporting their hypothesis that facial coloration provided important visual cues for individual recognition (Kohda et al. 2015). Follow-up research on *N. pulcher* using a familiar vs. unfamiliar fish-presentation protocol showed that these territorial fish indeed engaged in individual recognition of familiar fish, mollifying aggressive behavior (Saeki et al. 2018).

In another example of the importance of the head region, the ventral area around the eyes of damselfish (*Pomacentrus amboinensis*) exhibits subtle ultraviolet-reflecting pattern variation affording adequate cues for individual recognition. To study this effect, Parker and colleagues (2020) used food reinforcement to successfully train damselfish to discriminate high-contrast differences in facial patterns morphed from two actual face patterns. Such discrimination involved only the facial patterns and not the schemata of actual fish profiles (see also Siebeck et al. 2010 for initial research and Wang and Takeuchi 2017).

Pair-bonded pipefish (*Corythoichthys haematopterus*) can exhibit extraordinary individual recognition as characterized by their ritualized greeting behavior. Sogabe (2011, p. 195) postulates that individual variation in facial markings consisting of the number and position of brown spots on the snout might, in addition to speckles and ventral stripes on the body, contribute to visual recognition useful for distinguishing mates. A similar supposition regarding the perceptual aspects of the individual recognition of pair-bonded jewel fish was proposed by Noble and Curtis (1939), who were among the first researchers to document individual recognition by any teleost. In a study of schooling behavior, these researchers examined the early schooling behavior of 23 day-old jewel fish and the Central-American cichlid (*Cichlasoma cutteri*) and found rapid conspecific schooling indicative of evolved species recognition. Follow-up research showed that jewel-fish fry cross-fostered by *C. cutteri* until a juvenile age quickly schooled with conspecifics, providing further evidence suggesting that species recognition is guided by innate perceptual properties.

**Fig. 1:**
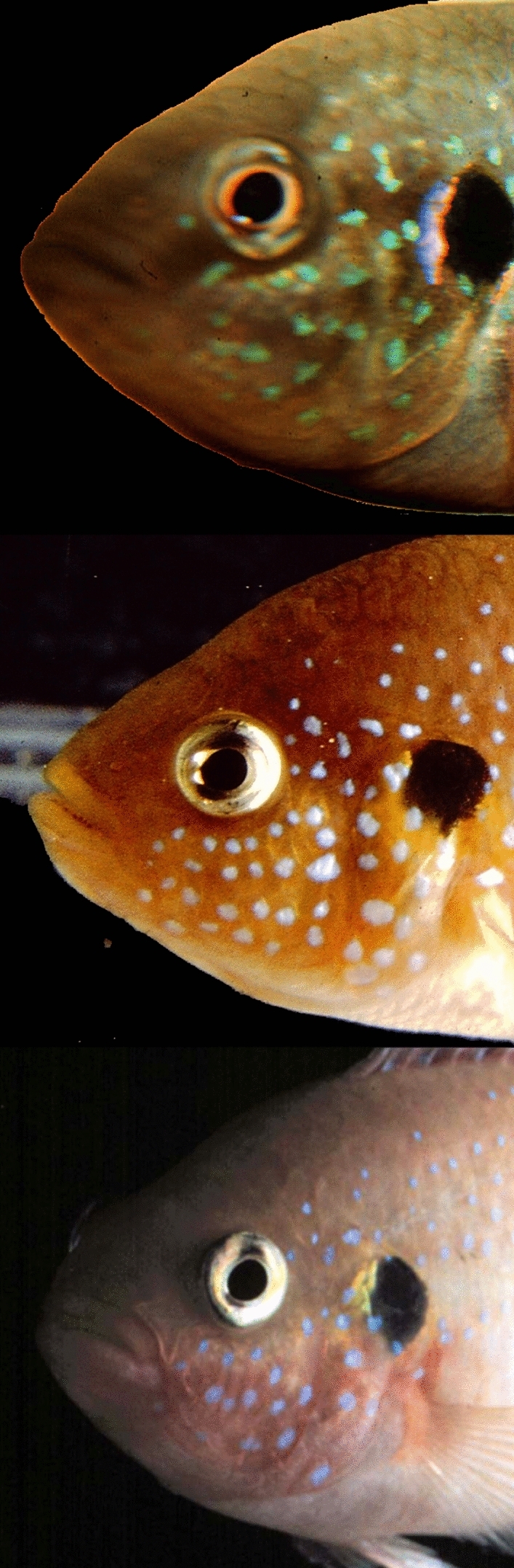
Examples of iridorpores covering the jewel fish face and operculum

At a more refined perceptual level involving learning, both jewel fish and *C. cutteri* adults pair-bond readily and engage in rapid mate recognition to defend against egg and fry in exchanging egg-guarding duties with their bonded mates. The head regions of both species show subtle pattern variation useful for individual recognition with jewel fish exhibiting a shimmering array of blue iridophores (Fig. 1). For *C. cutteri*, painting wider or narrowed bands of black lacquer across the top of the male’s head and snout does not preclude mate recognition by the female during egg-guarding exchanges. However, when an additional band is painted on one side of the head below the male’s eye, producing facial asymmetry, his mate will attack vigorously (Noble and Curtis 1939, p. 22). When this facial paint is removed and black longitudinal stripes are painted on the male’s sides, visually disrupting the vertical stripes along the body, mate recognition fails again. This exploratory finding suggests that the encompassing visual Gestalt of the head and body of *C. cutteri* is essential for mate recognition, a property restored when the body paint is removed.

## Respiration as a visual-attention measure

Since visual attention might characterize individual face recognition, the respiration rate of the fish was selected as the dependent measure for the current study. The frequency of respiration characterized by mouth and opercular movements is closely associated with heart rate (Taylor et al. 2009). These tightly coupled cardiorespiratory interactions can be modulated by the presence of potential threats or other disturbances. For example, cardio-deceleration and concomitant slowing of opercular movement can be induced by provocative light CS and shock US Pavlovian conditioning (cf. Otis et al. 1957; Woodard 1971; Schoel and Agranoff 1972; Scobie 1973). Moreover, because the eyeball is bordered by structures coupled with respiration, respiratory rhythmic movements can induce small eyeball movements causing retinal-image displacement (Ballintijn and Jüch 1984). Ballintijn and Jüch (1984, p. 106) further postulate for other teleosts that such retinal-image stabilization is essential for visual referencing of spatial position, facilitating sustained attention for prey catching.

The ventral portion of the mesencephalon appears to integrate visual and respiratory signals for retinal-image stabilization through feed-forward control. In carp (*Cyprinus carpio*) iontophoric delivery of horse radish peroxidase through recording electrodes has documented afferent projections from the optic tectum to the medullary respiratory system (Ballintijn et al. 1979; Luiten 1981). Unlike high-intensity respiration, normal respiratory movements do not activate eye muscles to compensate for eyeball displacement (Jüch 1982). In goldfish (*Carassius auratus*), respiration movements will suddenly cease for about 1 s when these fish detect a moving overhead shadow, with decelerated respiration continuing for several seconds after the apparent overhead threat has passed (Springer et al. 1977, p. 401). Follow-up overhead-shadow research by Laming and Savage (1981) showed that the increased arousal of goldfish measured by electroencephalograms was accompanied by decreases in heart and respiration rates. It is reasonable to argue here that a brief pause in respiration characterizes heightened goldfish attention by briefly eliminating mechanical displacement of the eyeball to achieve retinal-image stabilization that promotes retinal-image processing by the optic tectum. Such respiratory deceleration was predicted to occur in jewel fish when they engaged in individual face discrimination requiring sustained attention. Indeed, pilot research presenting a novel face model to jewel fish not used in the current experiment revealed initial closure of the operculum for about 1 s when the model was initially presented followed by slower respiration.

## Jewel fish face-recognition hypothesis

In their seminal research on individual recognition by jewel fish, Noble and Curtis (1939) focused their attention on mate recognition by females during their exchanges in egg guarding when both mates scrutinized each other closely on patrols. Using a painting protocol similar to that employed to study individual recognition by *C. cutteri*, Noble and Curtis found that painting only small parts of the jewel-fish head did not impact mate recognition whereas mate recognition usually failed when the both sides of the face were painted. In one example, painting the body but not the head with chrome yellow mixed with stopcock grease that roughly resembled jewel fish xanthoerythrophoric coloration, thereby concealing the longitudinal array of iridorphores, did not prevent mate recognition by egg-guarding females. However, when the male’s forehead, opercula, and area under both eyes were painted, the male was attacked immediately. This observation by itself arguably suggest that frontal views are important in jewel-fish mate recognition. Moreover, application of transparent stopcock grease without the paint to these facial areas did not preclude male face recognition. When the top of the male’s head was painted with three (1 cm dia.) spots of black lacquer the female continued to distinguish her mate from an unfamiliar male. However, the addition of more black spots on the sides of the male’s head led her to attack him, inferring again that the facial iridophore pattern was a fundamental visual cue for individual face recognition. Although suggestive of individual face recognition based on female aggressive behavior towards unfamiliar males, these unique jewel-fish observations by Noble and Curtis (1939) needed further documentation that the bluish iridophores indeed played an essential role in individual face recognition.

Our preliminary developmental research on jewel-fish face recognition, inspired by the findings of Noble and Curtis (1939), had hinted at the emergence of face recognition in 61 day-old socially reared juveniles that exhibited rapid habituation (non-associative response decline) to a repeated presentation of a jewel-fish face model displaying a realistic iridophore pattern. Habituation was illustrated by the sharp decline in escape behavior during the second model presentation (Tyler 1987, p. 137). Habituation was not evident in another group the same age that saw this model following by another model showing a different arrangement of iridophores. In the current research using scale models of jewel-fish faces with anatomically precise arrays of facial iridophores, we predicted that subadult jewel fish would discriminate familiar and unfamiliar iridophore patterns based on their respiratory behavior indicating elevated attention.

## Methods

### Construction of face models

Two schematic models of jewel-fish faces were constructed incorporating the anatomical distribution of iridophores visible from frontal views. To characterize typical individual variation in iridophore character numbers and spatial distribution, close-up photographs were initially taken of both sides of the head regions of adult jewel fish. Iridophore pattern photography was facilitated by restraining these fish in a net pressed against the aquarium wall. Adults from two spawns from different sets of parents were selected for anatomical analyses. In one group, there were 17 fish approximately 2 years old, yielding 7 photographs of the left side and 11 photographs of the right side. A second group of 13 adult subjects approximately 18 months old produced 12 right and 12 left lateral images. Thirty-two high-quality photographic images from each of the two sibling groups were projected onto a hexagonal grid that overlapped the lateral sides of each face. Hexagonal size approximated the diameters of the eyes and opercular eyespots. Counts were then made of iridophores in 10 hexagons visible in frontal views from regions surrounding the eyes and the mouth of both sides of the face (*n* = 8 fish/group) and coded as dependent measures for discriminant function analysis and group classification. Discriminant function analysis showed that only one fish was incorrectly classified, yielding (93.8%) for group membership. Two fish were then selected as exemplars for each of the two groups, based on having iridophore configurations closest to the group centroids, to create the iridophore layouts of the two face models used to examine face discrimination. One fish had hexagonal matrices with 16 and 17 iridophores for each side of its face while the other had 13 on each side of its face. In constructing the face models (Models 1 and 2), the layout of iridophores was equilibrated so each model displayed 15 iridophores on each side of its face either embedded in or removed from the largest grouping of iridophores.

**Fig. 2:**
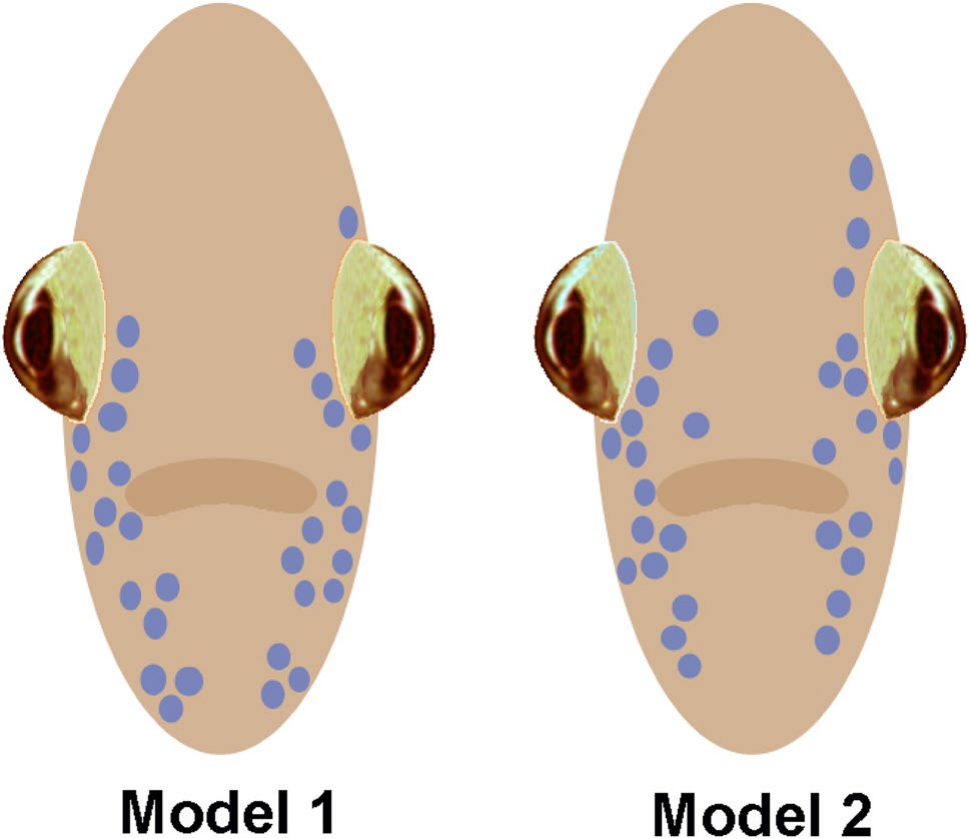
Illustration of face models exhibiting different arrangements of iridophores used to study face recognition in jewel fish

Models 1 and 2 were constructed of single ply card stock approximating typical jewel fish xanthoerythrophoric coloration (Munsell 2.5 YR 6/14), excluding the reddish ventral area of reproductively motivated adults. The model dimensions were 30 mm high and 13 mm wide and curved slightly in the horizontal plane to increase their realistic appearance. The bulbous eyes of the face models were angled backwards with laterally facing pupils. These simulated jewel-fish eyes were acquired from a craft shop for making hand-crafted toys. Blue reflective iridophores consisting of the dot-like centers of blue sequins were pasted on the models in the spatial arrangement illustrated in Fig. 2.

### Behavioral measurement

Each fish was used only once in a repeated-measures design. Because opercular opening and closing was difficult to measure accurately because fish could swim out of a close-up frame of analog black and white video, visual counts of opercular movement were used to quantify respiration change. To ensure that timing started uniformly across subjects according to their phase of respiration, models were moved into orthogonal alignment with the subject’s eyeball at a standard point in the respiration sequence. A digital stopwatch measured the elapsed time for 10 opercular movements that included the initial operculum closure as the first count. The accuracy of this counting method was evaluated by counting the oscillation frequency of a computer-generated circle-to-oval image for 10 s to simulate opercular opening and closing at about two beats per sec. This setup using a digital stopwatch for 18 trials generated a mean counting error of 30.1 ms (SD = 0.16, CV = 0.532%).

### Subjects and model presentations

Four groups of 189–190 day-old subadult jewel fish (4.5–4.7 cm length excluding tails), each consisting of nine randomly assigned fish, were tested for face-model discrimination. Fish were sampled alternately from larger groups held in separate aquaria, each consisting of 12–14 fish of mixed sex. Fish were reared and housed exclusively in three rack-mounted flat 73 l aquaria (51 × 59 × 24 cm), each equipped with 6 plastic houses with different graphical patterns to promote territorial behavior. Water temperature was maintained at 27 ± 2° C (pH 8.0) with a 12-h light–dark cycle. Aggressive territorial behavior (e.g., Coss and Globus 1978) was already evident prior to testing.

**Fig. 3:**
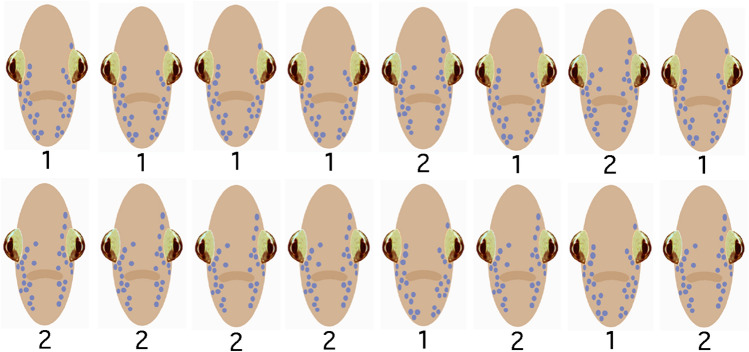
Left-to-right presentation sequence of models 1 and 2 for the two experimental groups

Two experimental groups were first given 3 preliminary model-presentation trials to induce rapid face-model habituation followed by 8 formal model-presentation trials used for statistical analyses. These groups were each presented either face-model 1 or face-model 2 for 6 of the 8 trials, with trials 1 through 4 presenting the same face models. During trials 5 and 7, however, the face models were switched to the novel models to examine any respiration changes (Fig. 3). Two control groups were each presented either model 1 or model 2 for all 8 trials without model switching.

**Fig. 4:**
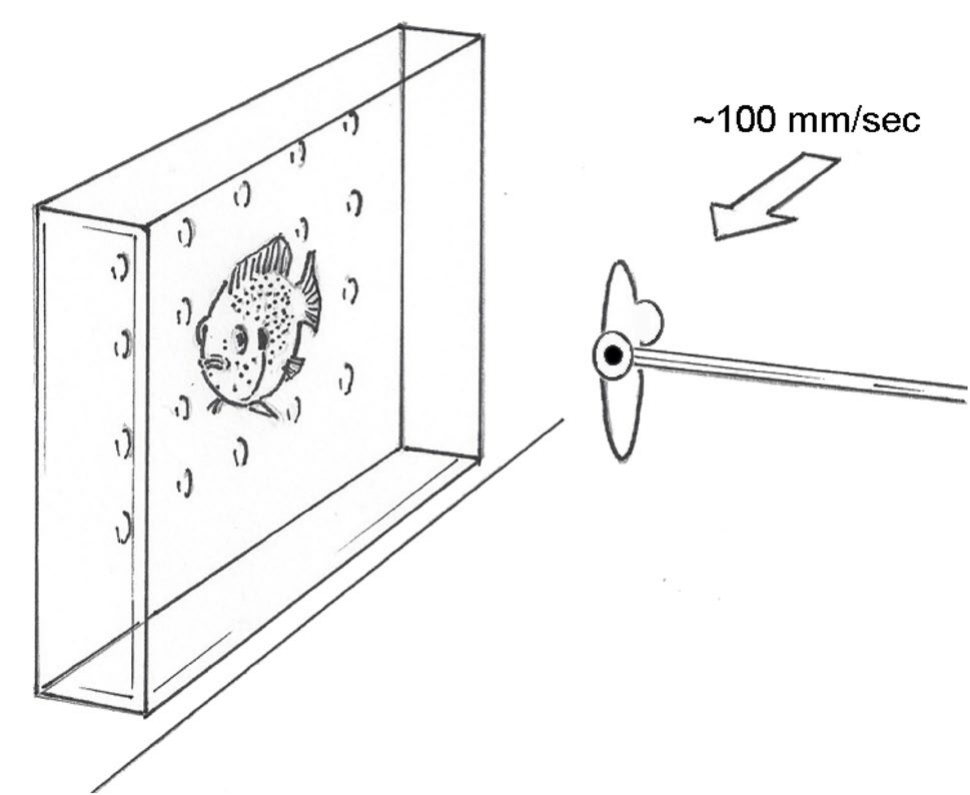
Illustration depicting the presentation of a face model to a jewel fish in a restraining compartment. Note the alignment of the model with the perceiver’s eye

**Fig. 5:**
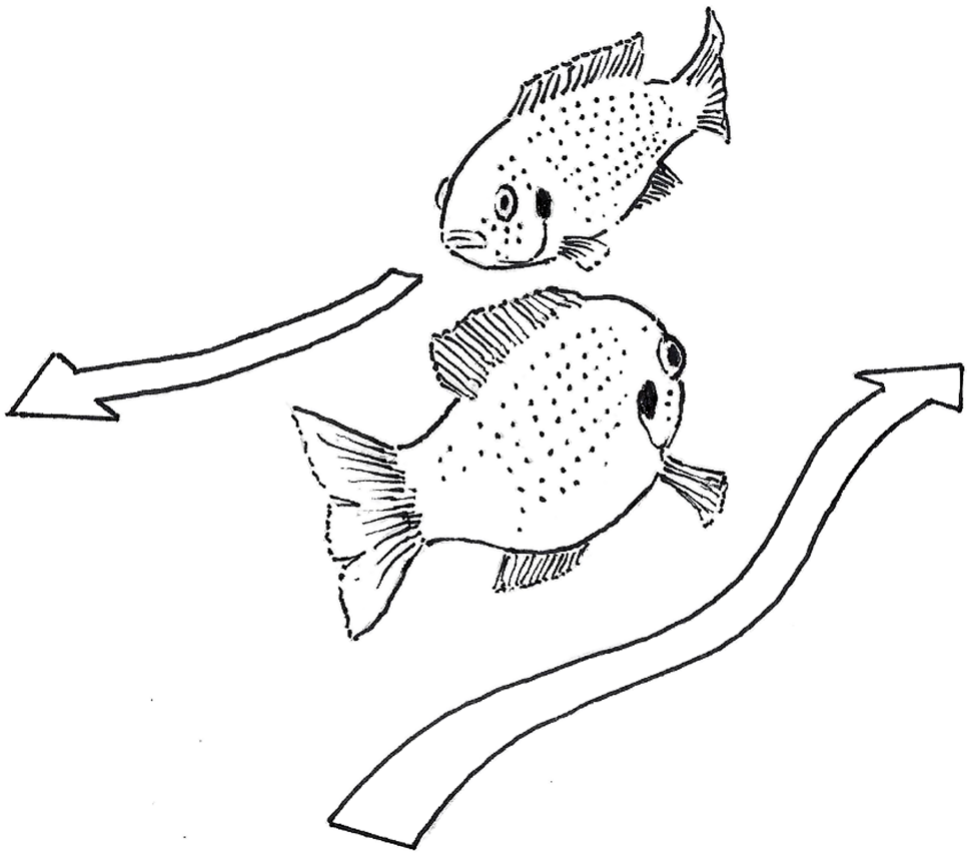
Typical trajectories of jewel fish swimming during face inspection when egg-guarding mates exchange guarding duties on patrol

To begin a face-discrimination test, a jewel fish was placed in a narrow plastic compartment (10.2 × 6.5 × 1.4 cm) supported by an aquarium floor that restricted lateral turning movement but not swimming in the other planes. Fish transferred from a holding aquarium into this restraining compartment using a small net were aroused initially, exhibiting a high respiration rate quantified during the 3 preliminary model-presentation trials discussed further below. To avoid startling our subjects, we deliberately presented our face models sideways into view to eliminate any intimidating looming effects. Additionally to prevent noise artifacts that could disturb the fish during model presentation, a model was mounted on a hand-held rod and moved silently with the right hand at ~ 100 mm/sec in the horizontal plane into the fish’s view, stopping at fish eye level 4 cm (± 2 mm) from the aquarium wall for the 3.5- to 6.5-s duration of 10 opercular beats (Fig. 4). This model-viewing distance is relatively consistent with the distance of typical face-inspection behavior between pair-bonded fish (see Fig. 5), and closer than the average frontal inspections of juvenile jewel fish (7.7 cm distance) measured from video (Coss 1978, p. 36). Each trial was initiated at approximately 30-s intervals. Laboratory lighting from overhead fluorescent fixtures illuminating the face model was 598 lx footcandles.

## Results

### Statistical analyses

As mentioned earlier, jewel fish were aroused physiologically after their transfer from their holding aquarium to the narrow model-presentation compartment that restricted lateral movement. This high arousal declined progressively during 3 preliminary presentations of the same face models used in trials 1 through 4 for the two experimental groups, as apparent from the increase in the average elapsed respiration times of 4.04 to 4.38 s for 10 opercular beats. Again, model presentations used the same procedure for all trials, irrespective of model switching. On the fourth model-presentation (trial 1 in the series of 8 trials), the average elapsed time of 10 opercular beats for the model switching and control groups increased to 4.72 s, approximating the elapsed respiration times for the control groups.

A mixed analysis of variance was applied to the data, consisting of a two-factor between subjects (model-switching and control groups; two models), one-factor within subjects (8 presentation trials) repeated-measures design. Only the main effect for trials, averaged for groups and models, was statistically significant (*F*_7,224_ = 2.627, *P* = 0.013). The interaction of groups and trials was also significant (*F*_7,224_ = 2.240, *P* = 0.032). The sources of this interaction were further evaluated by planned comparisons of specific trials to evaluate the theoretical hypothesis of face-model discrimination. Based on the elapsed time of 10 opercular beats, averaged for the two models and the model switching and control groups, pairwise comparisons of respiration changes during face-habituation trials 1 through 4 were not significant (M range = 4.732 s to 4.762 s). The same pairwise comparisons of respiration for face-habituation trials 1 through 4, averaged for the control groups, was also not significant (M range = 4.518 s to 4.712 s).

**Fig. 6:**
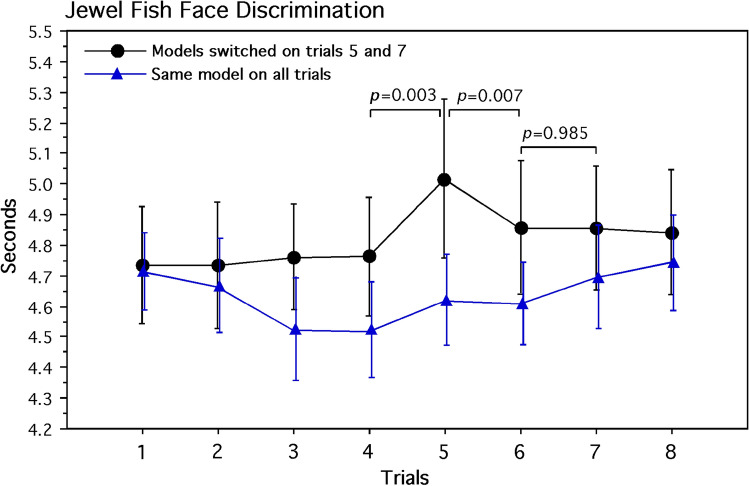
Average elapsed time for 10 opercular beats for models 1 and 2. Means and standard-error values are shown. For the experimental group, note the reliable decrease and increase in elapsed times when the habituated models are switched to the novel model in trial 5 and then switched back to the habituated models in trial 6. Switching the models again in trial 7 yielded a significantly similar decrease in elapsed time suggestive of one-trial habituation to the formerly novel faces

Support for our theoretical hypothesis of face-model discrimination was specifically evident for the comparison of the first model-switching episode, trial 5, presenting the novel face models compared with face-habituated models viewed in trials 4 and 6. This model-discrimination effect was not evident for the second model-switching episode, trial 7, compared with face-habituated trials 6 and 8 (Fig. 6). Averaged for the two model-switching groups, based on the timing of 10 opercular beats, jewel-fish respiration decelerated reliably from trial 4 presenting the habituated faces (M = 4.763 s, 95% CI 4.47 to 5.06 s) to trial 5 (M = 5.018 s, 95% CI 4.64 to 5.40 s) presenting the novel faces (*F*_1,32_ = 10.219, *P* = 0.003, *d* = 1.4). Conversely with switching back to the habituated faces during trial 6, jewel-fish respiration accelerated reliably (M = 4.856 s, 95% CI 4.59 to 5.12 s) compared with the novel face on trial 5 (*F*_1,32_ = 8.238, *P* = 0.007, *d* = 1.2). The quadratic trend for this decelerated and accelerated respiration (trials 4, 5, and 6), averaged for the two model-switching groups, was also significant (*F*_1,32_ = 11.893, *P* = 0.002), clearly documenting face-model discrimination during the first presentation of the two novel models. It is important to note here that the second model-switching episode from trial 6 (M = 4.608 s) to trial 7 (M = 4.696 s) showed no substantial change in respiration; and a planned comparison indicated that this mean-value similarity between the averages of the two habituated models and the two formerly novel models was significantly similar (1/*F*_32,1_ = 2500, *P* = 0.016). Such a result suggests a complete loss of face novelty during the second model-switching episode for both model-switching groups. This effect was further emphasized by the lack of a quadratic trend from trials 6, 7, and 8 that was not significant (*F*_1,32_ = 0.006, *P* = 0.941). A virtually flat trend in respiration during the second episode of model switching further suggests that face-model learning occurred with initial perception of the novel model, attenuating the need to suppress respiration for stabilizing the retinal image for evaluating the second presentation of the same face model. There were no reliable pairwise differences in respiration among trials 4 through 8 for the average of the two control groups (M range = 4.518 to 4.743 s) that experienced the same face models continuously.

**Fig. 7:**
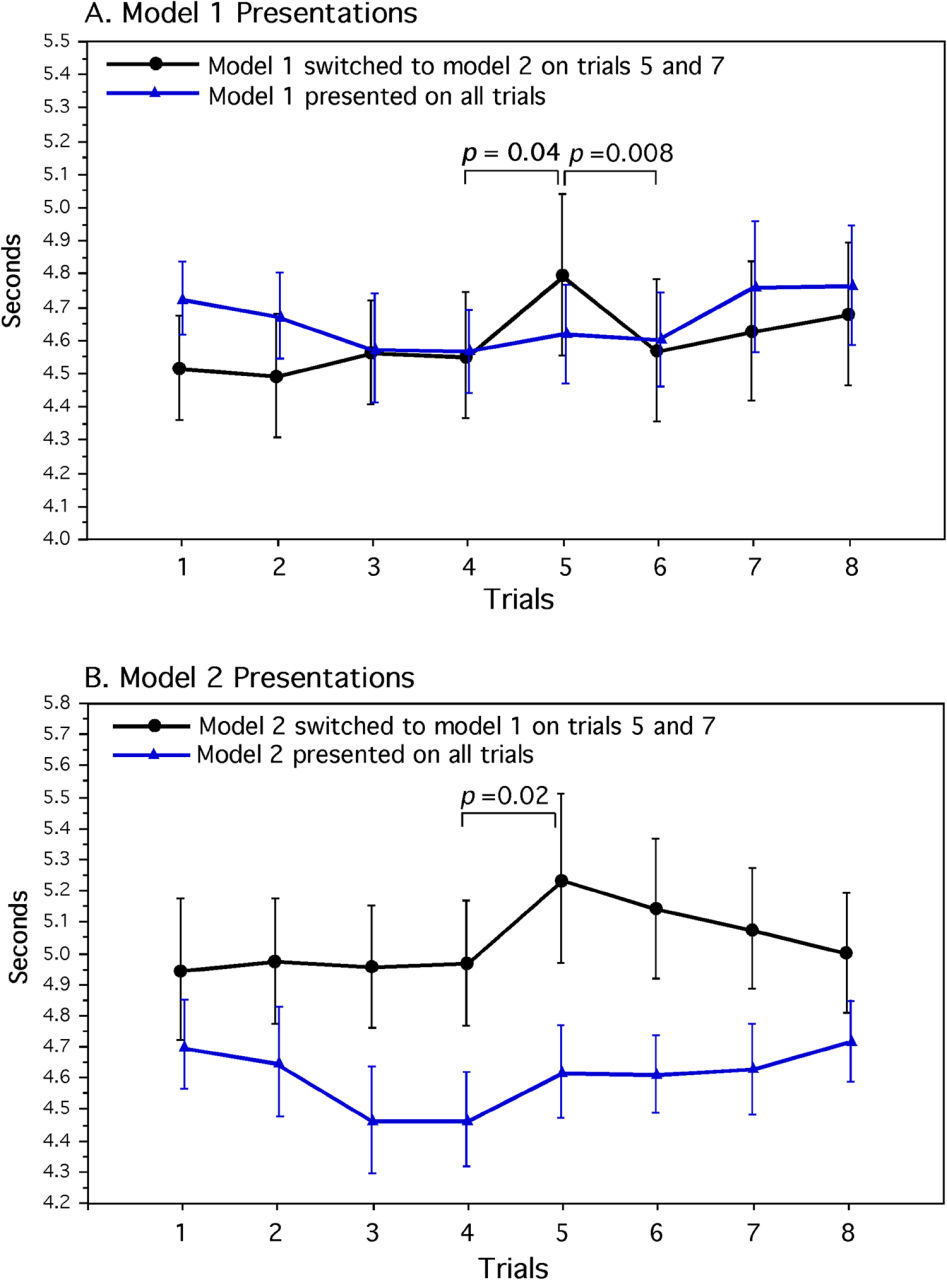
Average elapsed time for 10 opercular beats for the model-1 group **A** and model-2 group **B**. Means and standard-error values are shown. For both experimental groups, switching the habituated model to the novel model in trial 5 induced reliable increases is elapsed times for both groups. When the novels models are switched back to the habituated models during trial 6, only the model-1 group **A** showed a reliable decrease in elapsed time whereas the smaller decrease in elapsed time for the model-2 group **B** is suggestive of a carryover effect

Additional planned comparisons examined the effects of each face model separately for the model switching and control groups (Fig. 7). For the model-1 experimental group, switching from the habituated face model in trial 4 to the novel face-model 2 in trial 5 showed a reliable respiratory deceleration (*F*_1,32_ = 4.568, *P* = 0.040, *d* = 1.4). Switching back to the habituated face model in trial 6 also produced reliable respiratory acceleration (*F*_1,32_ = 8.042, *P* = 0.008, *d* = 1.6). The quadratic trend for respiratory deceleration and acceleration during trials 5, 6 and 7 was significant (*F*_1,32_ = 7.475, *P* = 0.010). Switching face models again during trials 6 and 7 and again from trials 7 and 8 did not engender reliable changes in respiration (M range = 0.456 to 0.440 s, respectively). The quadratic trend for respiratory changes for trials 6, 7 and 8 was not significant (*P* = 0.951). As in the above average for the two control groups, there were no reliable pairwise changes in respiration during trials 1 through 8 for the model-1 control group (Fig. 7A).

Similarly for the model-2 experimental group, presentation of the novel-face model 1 in trial 5 evoked reliable respiratory deceleration (*F*_1,32_ = 5.681, *P* = 0.023, *d* = 1.4). Unlike the model-1 group, however, the respiratory acceleration that occurred from the novel-model switch in trial 5 to the habituated face model in trial 6 was not significant (*F*_1,32_ = 1.496, *P* = 0.230). This finding suggest the presence of a carry over effect suppressing respiratory change following the switching of novel face-model 1 to the previously habituated face-model 2. Nevertheless, the quadratic trend of respiratory deceleration and acceleration during trials 4, 5 and 6 was again significant (*F*_1,32_ = 4.593, *P* = 0.040). The control group for model 2 that was presented this model continuously for all trials did not show any reliable pairwise changes in respiration during trials 1 through 8 (Fig. 7B).

## Discussion

Our prediction that two groups of jewel fish would distinguish different face models with distinct iridophore configurations was supported by their suppression of respiration when previously seen models engendering rapid habituation were switched initially to novel models. Unexpectedly, the second model-switching presentations yielded no substantial changes in the respiration of both groups, a finding indicating that model habituation can occur during one presentation trial. While the head region had been previously identified as important for mate recognition by jewel fish (Noble and Curtis 1939), our research pinpoints how differences in iridophore arrangements in exclusively frontal views can act as distinctive cues for discriminating different jewel-fish faces. Initial research by Tyler (1987, p. 137) had hinted at the emergence of face recognition in 61 day-old socially reared juveniles that habituated rapidly to a jewel-fish face model displaying a realistic iridophore pattern after two presentation trials. Again, model habituation, was not evident in another group that saw this model followed by a novel model showing a different arrangement of iridophores.

In territorial disputes among younger lab-reared juveniles, face-to-face confrontations are provocative, with the aggressors keeping a greater distance than when approaching from other angles (Coss 1978, p. 36). Nevertheless, the two-facing eyes of these models that are recognized innately (Coss and Globus 1978, 1979; Tyler 1987) would likely hold percipient attention during the suppression of respiration, engendering further assessment of the iridophore facial cues. This face-centering process with focused attention resulting from eye-schema detection would be analogous in process to the algorithms employed for face-pattern matching in artificial intelligence programs (cf. Seba and Kadyan 2013; Campadelli et al. 2009). Despite habituation to previously viewed models, detection of the discrepancy between these models and the switched novel models appears to have been very rapid as characterized by the initial pause in opercular closure during the counting of opercular-respiration beats.

Following initial face detection, we can speculate further on the pattern-recognition processes jewel fish employed based on our anatomical research on jewel-fish optic tectum (Coss and Globus 1978, 1979) and the extensive anatomical, electrophysiological, and brain-imaging research on other teleosts (e.g., O’Benar 1976; Meek 1983; Bollman 2019). Initially, the attention-drawing properties of the models’ two facing eyes coupled with their xanthoerythrophoric facial coloration would likely alert neurons in the optic tectum regulating eye movements (see Hermann 1971; Northmore 2017) that would sustain visual fixation on the models. By restricting retinal-image displacement via suppressed respiration, such augmented attention would foster iridophore-pattern scrutiny for pattern matching based on the iridophore organization in coordinate space, possibly anchored by both the model’s perimeter and the two-facing eyes as salient landmarks (for theoretical discussion of pattern matching, see Biederman 1987, p. 116).

In other species of fish, retinal images are projected topographically by retinal ganglion cells to the superficial and intermediate plexiform layers of the optic tectum (Fernald 1982; Del Bene et al. 2010; Preuss et al. 2014; Bollman 2019). We will now speculate from experimental evidence that the intermediate to deep layers of the jewel fish optic tectum facilitate the integration of image processing by tectal neural columns. For example, Coss and Globus (1979, p. 351) identified 12 different neuron types in the jewel fish optic tectum using rapid-Golgi staining, selecting for morphological analysis an interneuron that spanned deep to superficial tectal layers with numerous dendritic branches and spines. Adults reared in social isolation for more than a year exhibited fewer dendritic branches and spines like those of juveniles in the intermediate to deep tectal layers (*stratum griseum central* and *stratum periventriculare*) (cf. Coss and Globus 1978; Coss 1985, p. 268).

Of the remaining isolation-reared adults not used for histology, a one-year observation period of their social behavior in large aquaria, equipped with houses that ordinarily prompted territorial behavior, revealed that these isolates behaved more like juveniles by their failure to develop a dominance hierarchy typical of socially reared subadults (Coss and Globus 1979); Barnard and Burke (1979) theorize that individual recognition among contestants in dominance hierarchies might rely on asymmetries in fighting ability that are complemented by other assessment cues. Our interpretation that such a dominance hierarchy required individual recognition was bolstered further when isolation-reared breeding females guarding their eggs failed to recognize swapped male mates and continued to jointly guard the eggs. However, when this mate-swapping protocol was applied to socially reared jewel fish guarding their eggs, as in Noble and Curtis (1939, p. 21), fights broke out immediately after face inspection (unpubl. observ. 1979).

To summarize the broad implications of this developmental research and our current findings of face-model discrimination, the ability to distinguish models with two facing eyes from other eye-like arrangements is a fundamental property of jewel-fish face recognition (Coss and Globus 1978, 1979; Tyler 1987). Such discriminative flight from approaching models with two facing eyes persists in jewel-fish fry until the onset of territorial behavior, following which this flight response wanes to allow face-to-face confrontations during territorial disputes after older juveniles disperse from schooling (Chen et al. 1983). Such face-to-face confrontations in territorial juveniles would likely promote individual face learning; albeit, these older juveniles do maintain greater distances during face-to-face territorial confrontations compared with their harassment from diagonal, perpendicular, or caudal approaches (Coss 1978, p. 36). It is reasonable to propose here that the developmentally retarded interneural connectivity in the intermediate and deep layers in the optic tectum of isolates mirrored the neural complexity of younger, preterritorial juveniles and their inability to differentiate different arrangements of facial iridorphores at a level sufficient for individual face recognition (for 40 day-old juveniles, see Tyler 1987, p. 132).

In addition to jewel fish, evidence of individual face recognition in the cichlid (*Neolamprologus pulcher*) based on subtle variation in the shape of a facial bar and opercular eyespot (Kohda et al. 2015) and hierarchical social behavior suggestive of individual recognition by the cichlid, *Julidochromis transcriptus* (Hotta et al. 2015), argues strongly for additional study of the facial cues used to distinguish individuals. One method to test the range of jewel fish face-discrimination ability could employ high-definition video playbacks of individual jewel-fish siblings with similar arrays of facial iridiphores compared with individuals from different spawns with more distinctive arrays of iridophores. Additional behavioral research using video playbacks could entail complementary analyses of iridophores as recognition cues in pair-bonded mates using progressive masking of iridophores with xanthoerythrophoric-colored paint or by editing of high-resolution video playbacks to examine percipient attentional and aggressive behavior.

Although we have shown that jewel fish can differentiate facial-iridophore patterns, the functionality of iridophores needs to be explored further since most species of the genus *Hemichromis* exhibit reflective iridophores on their faces and bodies (see Loiselle 1992). One source of natural selection for reflective iridophores is water turbidity in West-African lakes and rivers that can vary seasonally as, for example, in the Bosumptwi lake, central Ghana and the Gambia River, Senegal (Simier et al. 2006; Shanahan et al. 2008). Future research could investigate mate recognition as a function of water transparency and suspended visible particulate density.

## Supplementary Information

Below is the link to the electronic supplementary material.

Supplementary file1 (XLS 47 KB)

## Data Availability

The raw data has been deposited at: https://osf.io/up6a3.

## Code availability

The codes have been deposited at: https://osf.io/up6a3.
